# Leeches as the intermediate host for strigeid trematodes: genetic diversity and taxonomy of the genera *Australapatemon* Sudarikov, 1959 and *Cotylurus* Szidat, 1928

**DOI:** 10.1186/s13071-020-04538-9

**Published:** 2021-01-12

**Authors:** Ewa Pyrka, Gerard Kanarek, Grzegorz Zaleśny, Joanna Hildebrand

**Affiliations:** 1 Department of Parasitology, Institute of Genetics and Microbiology, University of Wrocław, Przybyszewskiego 63, 51-148 Wrocław, Poland; 2 Ornithological Station, Museum and Institute of Zoology, Polish Academy of Sciences, Nadwiślańska 108, 80-680 Gdańsk, Poland; 3 Department of Systematic and Ecology of Invertebrates, Institute of Environmental Biology, Wrocław University of Environmental and Life Sciences, Kożuchowska 5b, 51-631 Wrocław, Poland

**Keywords:** Strigeidae, Leeches, Metacercariae, *Australapatemon*, *Cotylurus*

## Abstract

**Background:**

Leeches (Hirudinida) play a significant role as intermediate hosts in the circulation of trematodes in the aquatic environment. However, species richness and the molecular diversity and phylogeny of larval stages of strigeid trematodes (tetracotyle) occurring in this group of aquatic invertebrates remain poorly understood. Here, we report our use of recently obtained sequences of several molecular markers to analyse some aspects of the ecology, taxonomy and phylogeny of the genera *Australapatemon* and *Cotylurus*, which utilise leeches as intermediate hosts.

**Methods:**

From April 2017 to September 2018, 153 leeches were collected from several sampling stations in small rivers with slow-flowing waters and related drainage canals located in three regions of Poland. The distinctive forms of tetracotyle metacercariae collected from leeches supplemented with adult Strigeidae specimens sampled from a wide range of water birds were analysed using the 28S rDNA partial gene, the second internal transcribed spacer region (ITS2) region and the cytochrome* c* oxidase (COI) fragment.

**Results:**

Among investigated leeches, metacercariae of the tetracotyle type were detected in the parenchyma and musculature of 62 specimens (prevalence 40.5%) with a mean intensity reaching 19.9 individuals. The taxonomic generic affiliation of metacercariae derived from the leeches revealed the occurrence of two strigeid genera: *Australapatemon* Sudarikov, 1959 and *Cotylurus* Szidat, 1928. Phylogenetic reconstructions based on the partial 28S rRNA gene, ITS2 region and partial COI gene confirmed the separation of the *Australapatemon* and *Cotylurus* clades. Taking currently available molecular data and our results into consideration, recently sequenced tetracotyle of *Australapatemon* represents most probably *Au. minor*; however, unclear phylogenetic relationships between *Au. burti* and *Au. minor* reduce the reliability of this conclusion. On the other hand, on the basis of the obtained sequences, supplemented with previously published data, the metacercariae of *Cotylurus* detected in leeches were identified as two species: *C. strigeoides* Dubois, 1958 and *C. syrius* Dubois, 1934. This is the first record of *C. syrius* from the intermediate host.

**Conclusions:**

The results of this study suggest the separation of ecological niches and life cycles between *C. cornutus* (Rudolphi, 1808) and *C. strigeoides/C. syrius*, with potential serious evolutionary consequences for a wide range of host–parasite relationships. Moreover, phylogenetic analyses corroborated the polyphyletic character of *C. syrius*, the unclear status of *C. cornutus* and the separate position of *Cotylurus raabei* Bezubik, 1958 within *Cotylurus*. The data demonstrate the inconsistent taxonomic status of the sequenced tetracotyle of *Australapatemon*, resulting, in our opinion, from the limited availability of fully reliable, comparative sequences of related taxa in GenBank.
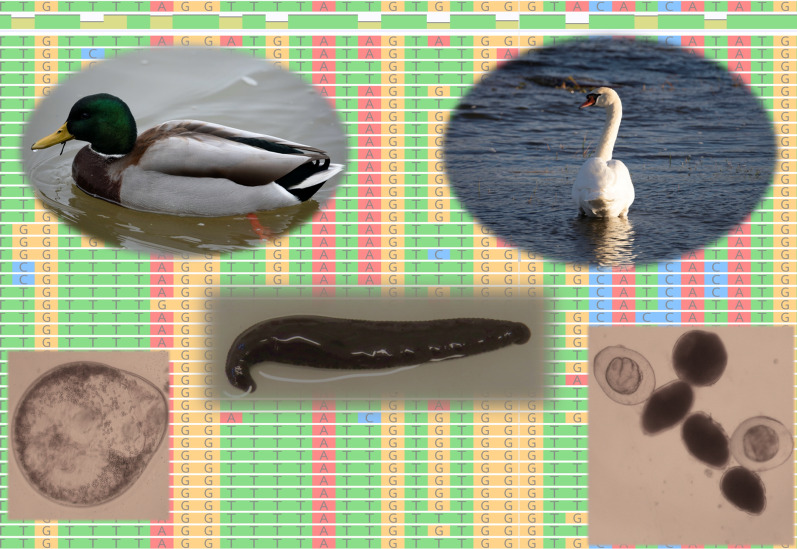

## Background

Leeches (Hirudinida) are an abundant and widely distributed group of aquatic invertebrates. Aside from their significance in freshwater ecosystems as prey and predators [1], leeches are the second intermediate hosts for some trematodes from the family Strigeidae Railliet, 1919 (genera: *Australapatemon* Sudarikov, 1959; *Cotylurus* Szidat, 1928) and *Cyathocotylidae* Mühling, 1898 (genus *Cyathocotyle* Mühling, 1898) [2–5]. These genera use prosobranch or pulmonate snails as their first intermediate hosts. Cercariae that are released from snails into the aquatic environment infect and develop in leeches to the invasive stage. Metacercariae (tetracotyle type in strigeid trematodes and prohemistomulum type in cyathocotylid trematodes) that develop in leeches are transmitted to the avian definitive hosts (a wide range of Anseriformes, but also recorded in Charadriiformes and Rallidae) by ingestion [5]. Despite the recognised role of leeches in the transmission of some digenean parasites in aquatic ecosystems, there remains an insufficient understanding of host–parasite relationships within this group of annelids, and only a few studies have analysed various taxonomic (e.g. [3–6]) and ecological (e.g. [7–12]) aspects of the occurrence and diversity of digenean larval stages in leeches. Moreover, contemporary understanding of the diversity of Strigeidae trematodes has been significantly changed by the recently discovered high level of interspecific and intergeneric homogeneity of morphological features related to an unexpectedly high level of genetic diversity, revealed within the genera *Australapatemon* and *Cotylurus* [13–16]. Given these developments, several various aspects of the ecology, taxonomy and phylogeny of the genera *Australapatemon* and *Cotylurus* require urgent, detailed study. Both genera possess a long and confusing history within Strigeidae. The genus *Australapatemon* was erected by Sudarikov [17] on the basis of the variability of the life cycles and structure of the cercarial protonephridial system observed in several species located previously in the genus *Apatemon* Szidat, 1928. In view of these differences, Sudarikov [17] redefined the genus *Apatemon* as being characterised by cercariae with ten flame cells and metacercariae encysted in fishes and erected the new genus *Australapatemon*, characterised by cercariae with 14 flame cells/protonephridia and metacercariae encysted in leeches. Since then, the taxonomic status and validity of these genera have been questioned and changed several times: some authors (e.g. [18, 19]) reduced *Australapatemon* to the level of a subgenus within *Apatemon*, while Yamaguti [20] restored it to the full generic rank, as further confirmed by Niewiadomska [21]. As the morphological differences between *Apatemon* and *Australapatemon* in both metacercariae as well as adult specimens are limited or subtle, these taxa have often been incorrectly determined (for details see [6] and references therein), and thus a few attempts to use molecular markers in the identification, taxonomy and phylogeny of these genera have been made in recent years [6, 13, 15, 22–24]. Importantly, a detailed molecular analysis of cercariae and adult specimens of *Australapatemon* sampled across North America clearly indicates the existence of several distinct lineages within this taxon and indisputably illustrates the hidden species diversity [13, 15]. In Europe, Huguenin et al. [25] identified another lineage within *Australapatemon* cercariae using Matrix-Assisted Laser Desorption-Ionisation–Time of Flight (MALDI-TOF) mass spectrometry, but the taxonomic position and molecular diversity of larval stages of *Australapatemon* collected from a wide range of intermediate hosts (snails and leeches) have not been the subject of extensive studies. The genetic variability and species richness within the genus *Australapatemon* from various geographical regions thus remain relatively unknown.

Another genus within Strigeideae that utilises hirudineans as hosts of invasive stages is the genus *Cotylurus*. This genus was erected by Szidat in 1928 for strigeid parasites of birds, and is characterised by vitellaria limited to the hindbody and a well-developed genital bulb. The metacercariae of *Cotylurus* occur in a wide range of snails and leeches [5]. In 1958, Bezubik [26] described a new species, *Strigea raabei*, based on trematode specimens collected from the bursa Fabricii of the garganey *Spatula querquedula* and ferruginous duck *Aythya nyroca* from eastern Poland, characterised by the presence of vitellaria both in the hind- and forebody (typical feature of the genus* Strigea*) and possession of a well-developed genital bulb (typical of the genus *Cotylurus*). Regarding these combinations of morphological features as unique, Sudarikov [27] erected the new genus *Cotylurostrigea* and placed in it two species: *Cotylurostrigea raabei* and *Cotylurostrigea strigeoides* Dubois, 1958. However, Dubois [19] treated the genus *Cotylurostrigea* as a synonym of *Cotylurus*, while Yamaguti [20] gave *Cotylurostrigea* subgeneric status within the genus *Strigea*. Using the results of a cladistics analysis based on morphological and ecological features, Zazornova and Sysoev [28] recognised *Cotylurostrigea* as a synonym of *Cotylurus*, similar to Niewiadomska [21] in the most recent system of Strigeidae. In 1969, on the basis of differences in life cycles and cercariae morphology, Odening [29] erected two new subgenera within the genus *Cotylurus*: (i) *Ichthyocotylurus*, characterised by cercariae with two pairs of penetration glands located behind the ventral sucker, metacercariae in fish intermediate hosts and fish-eating birds as final hosts; (ii) *Cotylurus*, including species with cercariae characterised by two pairs of penetration glands located in front of the ventral sucker, metacercariae encysted in gastropods and leeches and anseriform and charadriform birds as final hosts. Niewiadomska [30] regarded these taxa as valid and elevated both subgenera to the full generic rank, as confirmed in the most recent review [21]. However, representatives of *Cotylurus* show huge morphological variability, which led some authors to divide it into numerous subspecies with disputable validity [19], whereas others considered it as a polymorphic species with a wide host range and cosmopolitan distribution [31]. This led to basic problems with precise and adequate delimiting of particular species, which clearly indicates the need to use molecular techniques in studies on the taxonomy and phylogeny of *Cotylurus*. In recent years, Heneberg et al. [14] combined a morphological and molecular analysis of central European Strigeidae from avian definitive hosts to investigate the taxonomic position of several *Cotylurus* species and surprisingly revealed a high level of molecular diversity within morphologically well-established species. In Canada, Locke et al. [32] and Gordy and Hanington [15] also revealed the occurrence of several new species within this genus. These results clearly indicate the need for further, detailed morphological, molecular and phylogenetic studies.

Most of the recent molecular data on the taxonomy and structure of the genera *Australapatemon* and *Cotylurus* are based on free-living larval stages (cercariae), without a simultaneous analysis of the invasive stages in second intermediate hosts or a comparative molecular and morphological analysis of adults. Moreover, the majority of contemporary studies were based on simple molecular markers enabling a fully reliable comparison of the results with other data [15, 32]. In this article, we describe the identity and molecular diversity of tetracotyle metacercariae detected in four taxa of freshwater leeches (*Erpobdella octoculata*, *Glossiphonia complanata*, *Haemopis sanguisuga* and *Theromyzon tessulatum*), collected from three distinct localities in southern and northern Poland and based on several molecular markers (28S nuclear large ribosomal subunit gene, second internal transcribed spacer region [ITS2] ribosomal DNA [rDNA], mitochondrial [mt] cytochrome* c* oxidase subunit 1 [COI]). Our results were supplemented by data from the yet to be sequenced *Cotylurus* species, collected from avian hosts from northern Poland. On this basis, we present and discuss new data on the diversity and structure of the genera *Cotylurus* and *Australapatemon*. This study is the first comprehensive attempt to fully understand the identity and diversity of strigeid metacerariae from leech intermediate hosts in central Europe.

## Methods

### Host sampling protocols and necropsy procedures

**Fig. 1:**
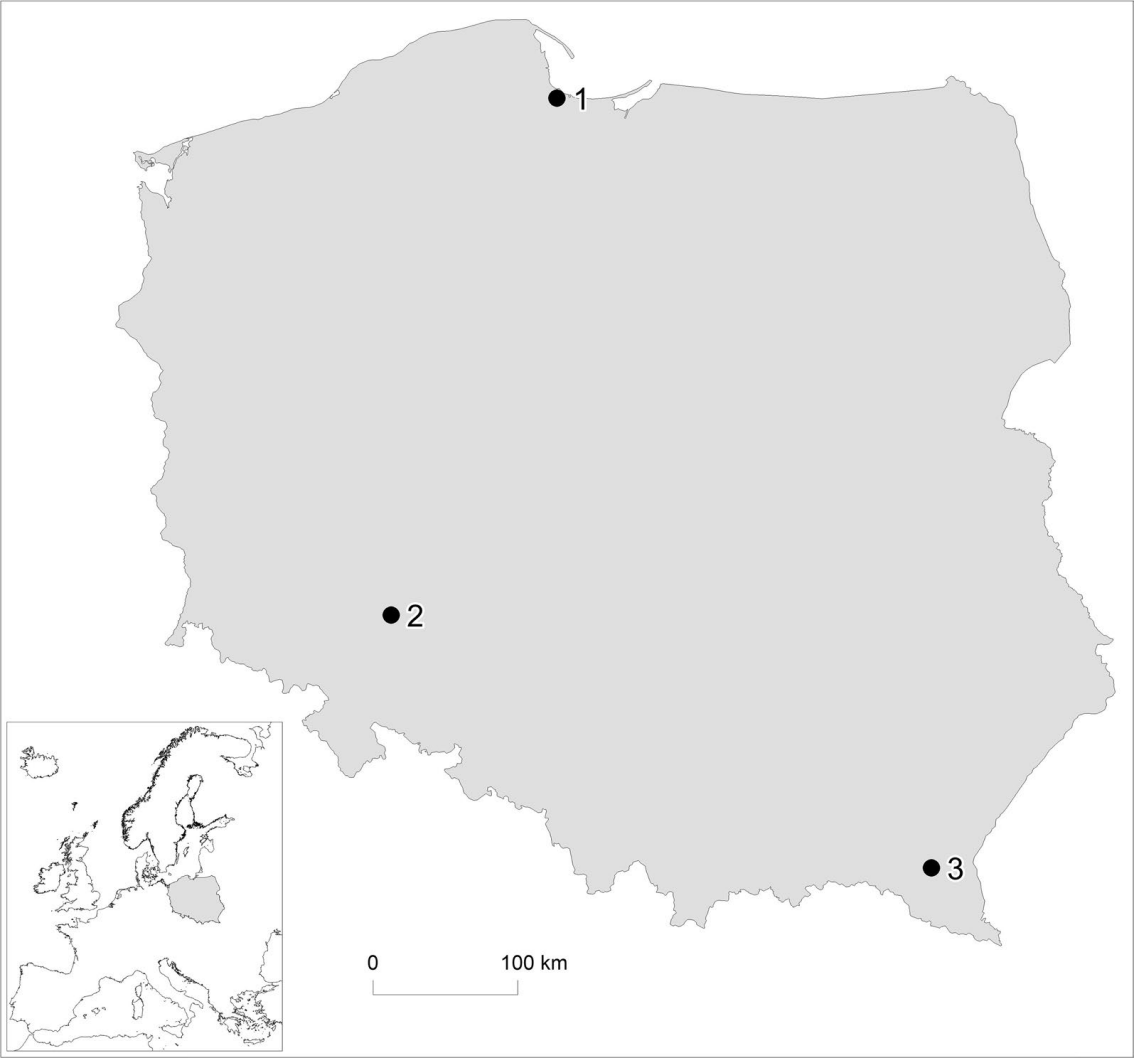
Map of the sampling stations:* 1* Gdańsk Pomerania,* 2* Lower Silesia,* 3* Subcarpathia Province

From April 2017 to September 2018, 153 leeches were collected from several sampling stations in small rivers with slow-flowing water and related drainage canals located in three regions of Poland: Gdańsk Pomerania, Lower Silesia and Subcarpathia Province (Table 1; Fig. 1). Leeches were collected manually, using entomological nets and home-made aluminium traps with beef liver or chicken hearts as bait. Additionally, some specimens were sampled by hand from littoral stones and bottom detritus. The leeches were transferred to a plastic box with water and adequate ventilation, transported to the laboratory and stored in the fridge. Before necropsy, leeches were identified to the species level based on morphological characteristics provided by Bielecki et al. [33]. Specimens of four species, namely *Erpobdella octoculata* (L., 1758), *Glossiphonia complanata* (L., 1758), *Haemopis sanguisuga* (L., 1758) and *Theromyzon tessulatum* Müller, 1774, were identified and examined for the presence of metacercariae (Table 1). In the case of infected leaches, taxonomic identification based on morphological features was confirmed by molecular results using the partial internal transcribed spacer (ITS) rRNA as a marker, and representative sequences were deposited in GenBank under no. MW256797 and MW256798. Leeches were initially anesthetised in 8% ethanol, then, after the cessation of movement, transferred to 70% ethanol for euthanasia [34], following which they were immediately opened longitudinally and the intestines separated from the skin and transferred to Petri dishes containing physiological sodium chloride solution where they were examined under a stereomicroscope. The distinctive forms of an unnamed tetracotyle metacercariae (both in pre-encystment stage and enclosed in an egg-shaped cyst) were extracted alive from the body cavity and mesenteries using preparation needles, washed in physiological sodium chloride solution, counted and fixed in hot 70% ethanol and finally preserved in the same medium for further processing. Material for further molecular analysis was randomly selected from the tetracotyle, according to the observed intensity of invasion: every 10th or 50th metacercariae (in the case of very low infection all metacercariae were sampled) from each infected host.

**Table 1:** Parasitological factors of leech infection

Localisation	Leech species	No. of necropsied/infected leeches	Tetracotyle prevalence (%)	Tetracotyle infection intensity (min–max; mean)	Recorded Strigeidae genus (no. of infected leeches/no. of leeches with mixed invasions)
Gdańsk Pomerania	*Haemopis sanguisuga*	66/51	77.3	1–235; 23.1	*Australapatemon* sp. (51/5)^a^
					*Cotylurus* sp. (5/5)^a^
Lower Silesia	*Erpobdella octoculata*	13/3	23.1	1–3; 2.0	*Australapatemon* sp. (2)
					*Cotylurus* sp. (1)
	*Haemopis sanguisuga*	37/8	21.6	1–15; 7.0	*Australapatemon* sp. (8/2)^a^
					*Cotylurus* sp. (2/2)^a^
	Total	50/11	22.0	1–15; 5.6	*Australapatemon* sp. (10/2)^a^
					*Cotylurus* sp. (3/2)^a^
Subcarpathia province	*Glossiphonia complanata*	8/0	–	–	–
	*Haemopis sanguisuga*	13/0			
	*Theromyzon tessulatum*	16/0			
	Total	47/0	–	–	–
TOTAL	*Erpobdella octoculata*	13/3	23.1	1–3; 2.0	*Australapatemon* sp. (2)
					*Cotylurus* sp. (1)
	*Glossiphonia complanata*	8/0	–	–	–
	*Haemopis sanguisuga*	116/59	50.9	1–235; 20.8	*Australapatemon* sp. (59/8)^a^
					*Cotylurus* sp. (8/7)^a^
	*Theromyzon tessulatum*	16/0	–	–	–
	TOTAL	153/62	40.5	1–235; 19.9	*Australapatemon* sp. (61/8)^a^
					*Cotylurus* sp. (9/8)^a^

^a^ Mixed invasions of *Australapatemon* sp. and *Cotylurus* sp. were detected

Several adult strigeid specimens collected from a wide range of water birds (mallard *Anas platyrhynchos*, gadwall *Anas strepera*, common pochard *Aythya ferina*, mute swan *Cygnus olor*, Eurasian coot *Fulica atra*) were used as reference material (Table 2). After isolation from the gastrointestinal tracts of the definitive avian hosts, the digeneas were rinsed in physiological salt solution, initially identified alive under the microscope and then fixed in hot 70% ethanol for further morphological and molecular analyses. The selected digeneans were then stained with alcohol borax carmine, dehydrated in ethanol series, cleared in clove oil and mounted in Canada balsam. Voucher specimens of adult flukes are deposited in the Polish Collection of Parasitic Helminths, Museum of Natural History, Wrocław University, Poland with collection numbers 204290 (*Apatemon fuligulae* Yamaguti, 1933), 204291–204292 [*Cotylurus cornutus* (Rudolphi, 1808)], 204293–204297 (*C. hebraicus* Dubois, 1934), 204298–204302 [*C. raabei* (Bezubik, 1956)], 204303–204307 (*C. strigeoides* Dubois, 1958) and 204308–204315 (*C. syrius* Dubois, 1934).

**Table 2:** List of sequences of strigeids obtained in this study

Digenean taxa	Host species and geographic region of Poland	Life cycle stage^a^	Own number/GenBank number		
			COI	28S	ITS
*Apatemon fuligulae*	*Aythya ferina* Gdańsk Pomerania	A	S3/ MW204830	S3/MW244636	S3/MW244656
*Australapatemon* sp.	*Anas strepera* Gdańsk Pomerania	A	1809/MW204820	1809/MW244635	1809/MW244655
*Australapatemon* sp.	*Haemopis sanguisuga* Gdańsk Pomerania	M	PG1/MW204826 PG2/MW204827 PG4/MW204828 PG5/MW204829	PG1/MW244633 PG2/MW244634 – –	PG1/MW244650 PG2/MW244651 – –
*Australapatemon* sp.	*Haemopis sanguisuga* Lower Silesia	M	PS2/MW204821 PS3/MW204822 PS4/MW204823 PS5/MW204824 PS7/MW204825	PS2/MW244630 PS3/ MW244631 – – –	– PS3/ MW244654 – PS5/ MW244653 –
*Australapatemon* sp.	*Erpobdella octoculata* Lower Silesia	M	–	PS11/MW244632	PS11/MW244652
*Cotylurus cornutus*	*Anas platyrhynchos* Gdańsk Pomerania	A	S10/MW204806	S10/MW244637	S10/MW244657
*Cotylurus hebraicus*	*Fulica atra* Gdańsk Pomerania	A	1443/MW204805 –	1443/MW244638 1492/MW244639	1443/MW244667 1492/MW244668
*Cotylurus raabei*	*Anas platyrhynchos* Gdańsk Pomerania	A	S2/MW204804	S2/MW244649	S2/MW244669
*Cotylurus strigeoides*	*Anas platyrhynchos* Gdańsk Pomerania	A	S9/MW204807	S9/MW244640	S9/MW244658
*Cotylurus syrius*	*Cygnus olor* Lower Silesia	A	LD/MW204819	LD/MW244648	LD/MW244666
*Cotylurus syrius*	*Cygnus olor* Małopolska Region	A	LK/MW204818	LK/MW244647	LK/MW244665
*Cotylurus* sp.	*Haemopis sanguisuga* Gdańsk Pomerania	M	PG3/MW204814 PG6/MW204816 PG8/MW204815 PG9/MW204817	PG3/MW244644 – PG8/MW244645 PG9/MW244646	PG3/MW244661 – PG8/MW244660 PG9/MW244664
*Cotylurus* sp.	*Haemopis sanguisuga* Lower Silesia	M	PS1/MW204808 PS3/MW204809 PS5/MW204810 PS8/MW204811	PS1/MW244641 – PS5/MW244643 –	PS1/MW244659 – PS5/MW244662 –
*Cotylurus* sp.	*Erpobdella octoculata* Lower Silesia	M	PS10/MW204812 PS12/MW204813	PS10/MW244642 –	PS10/MW244663 –

*COI*, Cyclooxygenase gene; ITS, Internal transcribed spacer region

^a^A, Adult; C, cercaria; M, metacercaria

The ecological terms used in this study are those defined by Bush et al. [35].

### Molecular analysis

DNA was extracted from single, alcohol-fixed metacercariae and adult worms using a commercial kit (DNeasy Blood and Tissue kit; Qiagen, Hilden, Germany), according to the manufacturer’s protocol. PCR amplification of 28S) and the ITS as well as of the mitochondrial gene encoding COI was carried using the KAPA2G Robust HotStart ReadyMix (Sigma-Aldrich, St. Louis, MO. USA) and primers selected based on the literature. A list of primers and the cycling conditions of the PCR reaction are presented in Additional file 1: Table S1.

The PCR results were visualised following electrophoresis in a 1% agarose gel. The electrophoresis products were purified using the Exo-BAP kit (EURx) or QIAquick Gel Extraction kit (Qiagen) when non-specific products were present. Purified products were sequenced directly in both directions using the PCR primers. Contiguous sequences were assembled using Geneious software (Geneious 9.1.8; https://www.geneious.com). The representative sequences were submitted to GenBank under accession numbers presented in Table 2. The alignments included newly obtained sequences and closely related representatives of Strigeidae currently available in GenBank (Additional file 1: Table S2) and were prepared using ClustalW multiple alignment implemented in MegaX [36]. Sequences of the 28S rDNA partial gene, ITS2 region and the COI fragment were aligned in three independent datasets. Phylogenetic analyses were conducted using Bayesian inference criteria as implemented in MrBayes ver. 3.2.7 software [37] and were run on the three datasets individually. The general time-reversible model with estimates of invariant sites and gamma distributed among-site variation (GTR + I + G) was identified as the best-fitting nucleotide substitution model for 28S and COI, and the Hasegawa–Kishino–Yano substitution model with gamma distributed among-site variation (HKY + G) for ITS2, using jModelTest 2 software [38]. The consensus trees were visualised in FigTree ver. 1.4.4 software [39] and annotated in CorelDraw® (Corel Corp., Ottawa, ON, Canada).

We also used GMYC analysis (the Generalised Mixed Yule Coalescent Model) as a tool for species delimitation [40, 41]. This method works for a single locus tree; we used the COI sequence to construct an ultrametric tree with BEAST v. 2.4.4 [42]. Prior to analysis the alignment was collapsed to unique haplotypes and the outgroupvremoved. Thus, the GMYC analysis contained 34 haplotypes, the nucleotide substitution model was set to HKY + G and we used the coalescent model with constant population size (which is the most appropriate for modeling the relationships among individuals from the same species) with strict clock. GMYC analysis was done in R software (R v. 4.0.2; R Foundation for Statistical Computing, Vienna, Austria) with the following packages: ‘ape’, ‘paran’, ‘rncl’ and ‘splits’.

## Results

### Parameters of infection

Of the the 153 leeches investigated, metacercariae of the tetracotyle type (both encysted and non-encysted) were detected in the parenchyma and musculature of 62 specimens (overall prevalence 40.5%) from two localities: Gdańsk Pomerania (51 infected among 66 *H. sanguisuga* investigated) and Lower Silesia (3 infected among 13 necropsied specimens of *Erpobdella octoculata* and 8 infected among 37 *H. sanguisuga*); none of the specimens of *G. complanata* and *T. tessulatum* were infected (Table 1). The highest prevalence of metacercariae was observed among leeches collected from Gdańsk Pomerania (Table 1). Leeches sampled in Subcarpatia Province were not infected. Regarding host species, the highest prevalence was observed in *H. sanguisuga* (59 infected, prevalence 50.9%).

The highest mean intensity was detected among leeches from Gdańsk Pomerania [23.1 individuals (ind.)]; a much lower mean prevalence was recorded in Lower Silesia (5.6 ind.). Regarding host species, the highest mean intensity was noted in *H. sanguisuga* (20.8 ind.) (Table 1).

### Molecular identification of detected metacercariae and phylogenetic analyses

The taxonomic generic affiliation of encysted and non-encysted metacercariae derived from leeches was conducted based on BLAST (Basic Local Alignment Search Tool) comparison of sequences of 28S rDNA and resulted in confirmation of the occurrence of two strigeid genera: *Australapatemon* and *Cotylurus*.

Although comprehensive molecular studies of the structure of the Strigeidae are rather rare in the literature, a few taxonomic studies concerning the systematic position of several genera within this family have been published recently [6, 14]. Based on the results of these studies, and given the main aim of the current study (identification and molecular diversity of strigeid metacercariae in leeches from central Europe), the structure of our datasets was determined first of all by the availability of sequences from previously published European isolates. Thus, the presented analyses are focussed mainly on the identity, molecular diversity and phylogenetic relationships within two genera from which larval stages were detected in leeches: *Australapatemon* and *Cotylurus*. As the sequences of *Australapatemon* and *Cotylurus* published previously by Heneberg et al. [14] constitute a significant part of the comparative material necessary for establishing the taxonomic position of the recently collected and analysed materials, we used the same parts of ITS region and COI gene, and also the alignments used for our phylogenetic analyses were trimmed to the length used in the cited work [14], i.e. 270 bp for ITS2 and 295 bp for COI. However, full-length received alignments were deposited in GenBank. The length of the 28S alignment was 980 bp and included almost all sequences of *Australapatemon* and *Cotylurus* available in GenBank, with the exception of that of *C. gallinulae* (length 700 bp). Phylogenetic reconstructions of the partial 28S rRNA gene, ITS2 region and partial COI gene clearly confirmed the separation of the *Australapatemon* and *Cotylurus* clades. The obtained results do not reveal any host–parasite specificity within detected tetracotyle of *Australapatemon* and *Cotylurus* and their leech hosts (Table 1).

#### *Australapatemon*

**Fig. 2:**
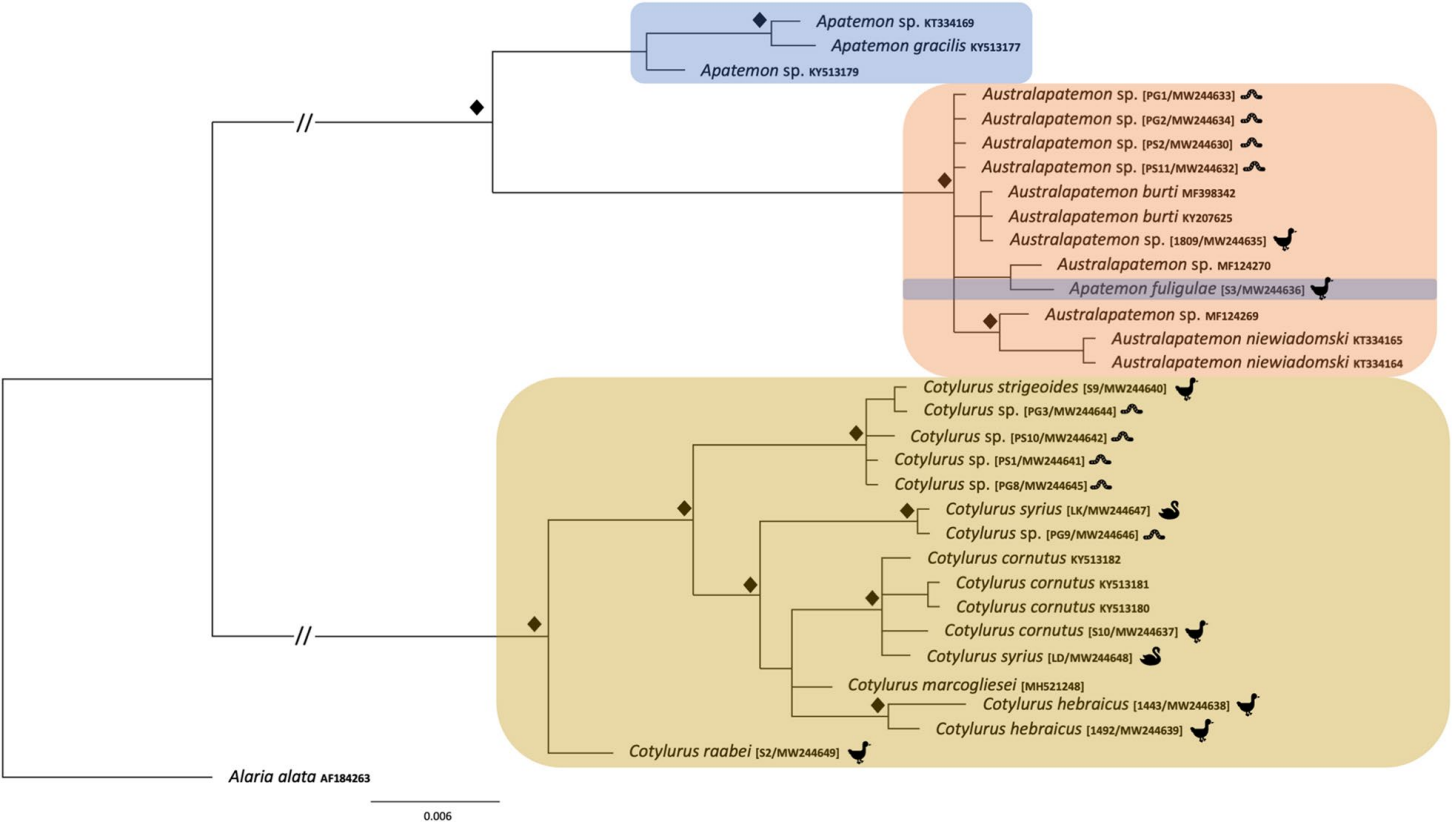
The phylogenetic relationships of the *Australapatemon* and *Cotylurus* species. Analysis of the 28S nuclear large ribosomal subunit gene (28S) marker based on Bayesian inference. Diamond symbol indicates a posterior probability of > 90%. The pictograms placed at the names of sequences obtained in the present study reflect the host species

The genetic divergence between *Australapatemon* specimens obtained from leeches in the present work was considered negligible based on the 28S sequences and the absence of intraspecific variability within localities (Fig. 2). These sequences clustered in a well-supported clade with sequences obtained from trematodes identified as *Australapatemon burti*, isolates from an adult fluke from Mexico (MF398342) and cercaria from Canada (KY207625), larvae identified as *Australapatemon* sp. from Canada (MF124269, MF124270) and the adult form of *Au. niewiadomski* from New Zealand (KT334164, KT334165) (Fig. 2). The similarity of new sequences with the sequences mentioned above ranged from 99.9% (1-nucleotide difference) for *Au. burti* to 99.4% for *Au. niewiadomski* (6-nucleotide difference). The sequence of an isolate identified as *Australapatemon* sp. obtained from *Anas strepera* showed 100% homology with previously published sequences of *Au. burti* KY207625 and MF398342 (based on 1244 and 1207 bp, respectively).

**Fig. 3:**
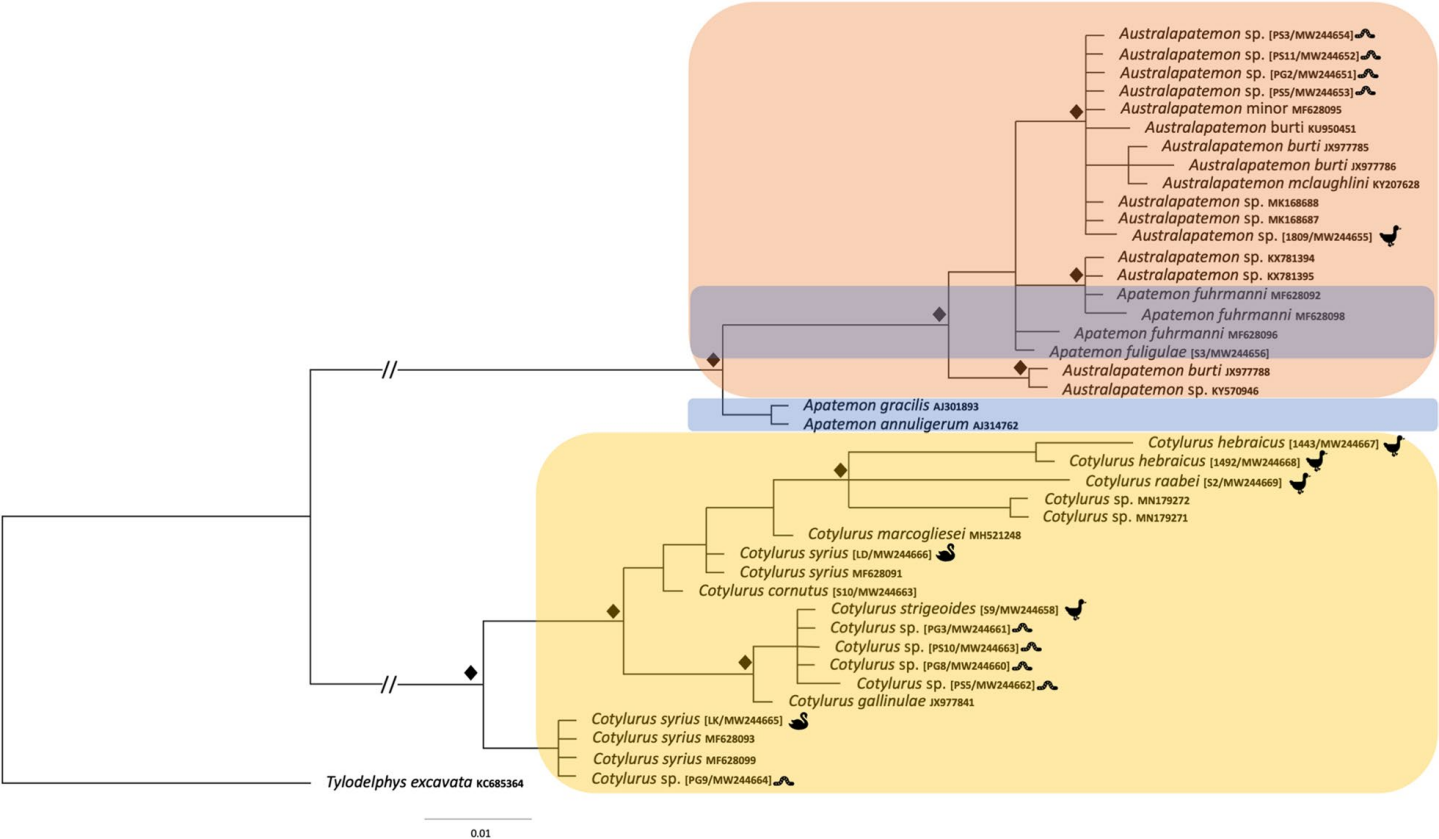
The phylogenetic relationships of the *Australapatemon* and *Cotylurus* species. Analysis of the second internal transcribed spacer region (ITS2) rDNA marker based on Bayesian inference. Diamond symbol indicates a posterior probability of > 90%. The pictograms placed at the names of sequences obtained in the present study reflect the host species

The DNA sequences of the ITS2 fragment from metacercariae sampled from leeches in the present study showed 100% identity among themselves and with sequences of *Au. minor* from *Anas platyrhynchos* from the Czech Republic (MF628095) and *Au. burti* from cercariae from Slovakia (KU950451) and Canada (KY207626), as well as with an isolate from the USA described as *Australapatemon* sp. (KY570947). These sequences formed one clade with trematodes identified as *Au. mclaughlini* (1-nucleotide difference in comparison with isolates from leeches) and *Au. burti* from Mexico (2-nucleotide difference) (Fig. 3).

**Fig. 4:**
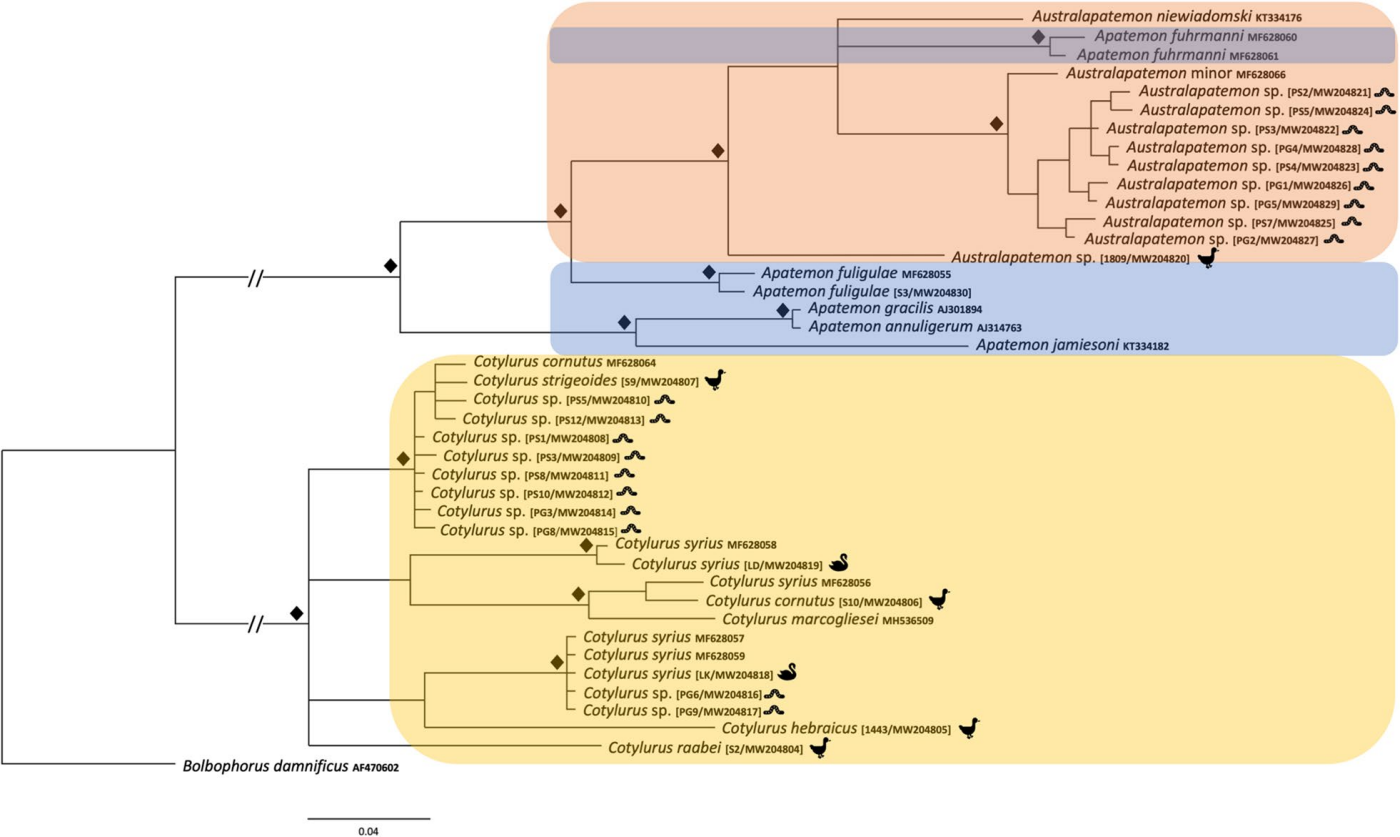
The phylogenetic relationships of the *Australapatemon* and *Cotylurus* species. Analysis of the cytochrome* c* oxidase subunit 1 (COI) mtDNA marker based on Bayesian inference. Diamond symbol indicates a posterior probability of > 90%. The pictograms placed at the names of sequences obtained in the present study reflects the host species

Newly generated COI sequences from metacercariae collected from leeches formed a well-supported clade with *Au. minor* (MF6280066) within the *Australapatemon* branch (Fig. 4). Unfortunately, there are no available sequences of *Au. burti* that could be added to this analysis; thus, we were unable to indisputably determine the final taxonomic affiliation of isolates from leeches.

Our phylogenetic analyses carried out separately based on three genetic markers demonstrated with strong support the sister relationship among *Australapatemon* and some species of *Apatemon*: *A. gracilis* and *Apatemon* sp. “*jamiesoni*” (Fig. 2). However, the taxonomic position of *Ap. fuligulae* remains unclear: in phylogenetic reconstructions derived from the 28S and ITS2 datasets this species nested between *Australapatemon* isolates, but according to the result of the COI analysis *Ap. fuligulae* from Poland and the Czech Republic clustered with *Apatemon* sp. “*jamiesoni*”, although without strong support. Simultaneously, sequences of *Apatemon*/*Australapatemon fuhrmanni*, the generic affiliation of which was discussed by Heneberg et al. [14], were located inside the *Australapatemon* clade generated based on ITS2 and COI dataset analyses (Figs. 3, 4).

#### *Cotylurus*

The newly generated sequences of 28S rDNA, the ITS2 region and COI mtDNA derived from metacercariae obtained from leeches fall in two well-supported lineages corresponding to *Cotylurus strigeoides* Dubois, 1958 (isolate from *Anas platyrhynchos*) and *C. syrius* Dubois, 1934 (100% homology with isolates from *Cygnus olor* from Poland and the Czech Republic; accession numbers MF628093, MF628099 for ITS2 and MF628057, MF628059 for COI). The intraspecific genetic divergence between sequences in clades grouping metacercariae and the adult form of *C. strigeoides* ranged from 0 to 0.2% for the 28S dataset, from 0 to 1.5% for the ITS2 dataset, and from 1 to 1.7% for the COI dataset.

The analysis of 28S rDNA and COI sequences showed the distinct position of *Cotylurus raabei*, while other members of the genus *Cotylurus* formed the clade within which *C. strigeoides* appeared as a sister lineage to the group, including *C. cornutus* (Rudolphi, 1808), *C. syrius*, and recently obtained sequences of *C. hebraicus* Dubois, 1934. Moreover, all three phylogenetic analyses corroborated the polyphyletic character of *C. syrius* and also showed the unclear status of *C. cornutus* (Figs. 2, 3 and 4). Two recently obtained isolates of adult trematodes from *Cygnus olor* were located within two separate lineages of *C. syrius*, but two isolates from metacercariae were located within only one of them.

The sequence of 28S rDNA from *C. cornutus* obtained in the present study (adult trematode from *Anas platyrhynchos*) clustered together with isolates determined as *C. cornutus* derived from metacercariae from Norway (KY513180–KY513182) with similarity of 99.6–99.7%. However, due to the lack of comparative sequence material for the ITS region and COI mtDNA and the ambiguous position of our *C. cornutus* isolate in the trees generated based on the above datasets (Figs. 3, 4), the taxonomic position of this species remains unclear.

### GMYC results

**Fig. 5:**
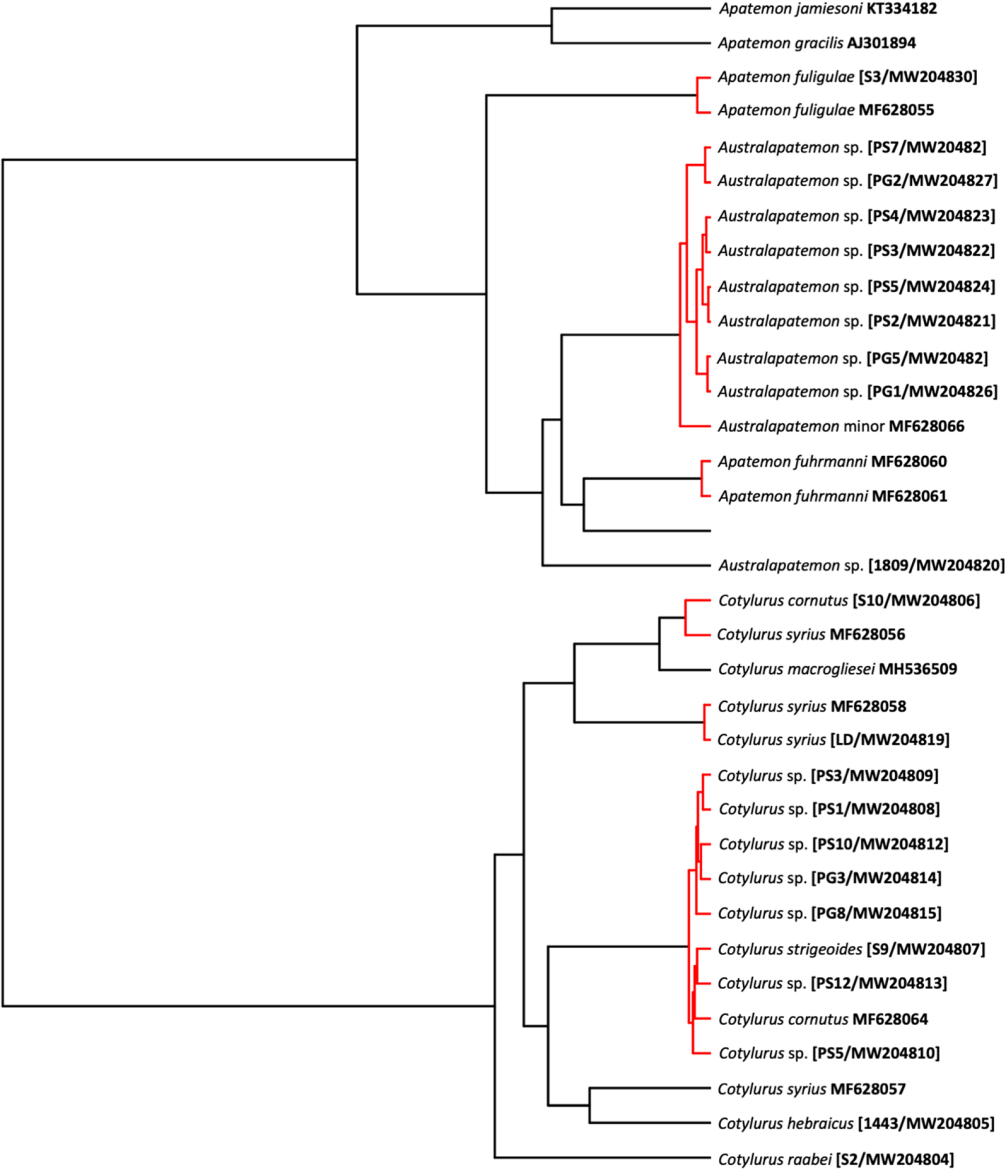
The Generalised Mixed Yule Coalescent Model (GMYC) analysis. Red lines indicate intraspecific variation, black lines show between species branches

Within the 34 haplotypes GMYC analysis revealed the presence of 14 species and generally confirmed topology of clades derived by Bayesian analysis (Fig. 5). It would appear that all larval forms of strigeid flukes obtained from leeches could be matched to three species, i.e. *Australapatemon minor* and *Cotylurus strigeoides* and *C. syrius* (not shown in the tree as it had an identical haplotype to the GenBank sequence). However, in the case of the *C. strigeoides* and *C. syrius* clade, GMYC support reached 86% while for the *Au. minor* clade it was 51%.

## Discussion

The results of this study provide several new and important insights into the diversity of strigeid trematodes occurring in freshwater leeches in central Europe and clearly emphasise the importance of this group of aquatic invertebrates in the circulation of trematodes in the aquatic environment. The observed prevalence of tetracotyle metacercariae detected in leeches (40.5%) was relatively high compared to that reported from other studies conducted in central and eastern Europe: during investigations of parasites of species-rich and diverse communities of leeches in the eutrophic Drużno Lake (northern Poland), Dobrowolski [3] detected larvae of strigeid and cyathocotylid trematodes among 20% of the leaches analysed, while in lakes of Kazakhastan, Zhatkanbaeva [43] and Zhatkanbaeva and Akhmetova [44] detected tetracotyle metacercariae in 19.4% of the investigated leeches. On the other hand, studies from eastern Europe have revealed the prevalence of tetracotyle metacercariae at much higher levels: in the Volga estuary Sudarikov et al. [45] found metacercariae in 60% (75 of 125) of the leeches analysed, and Rajshite [46] detected strigeid metacercariae in 53% of leeches from the Volga and Niemen estuaries.

Tetracotyle metacercariae usually classified as *Tetracotyle typica* de Fillippi, 1855 have been widely recorded from snails and leeches in Europe, but their true taxonomic status has remained unknown for many years. Meanwhile, the adult form of *T. typica* sampled from snails has been identified as *Cotylurus cornutus* (Rudolphi, 1808) (for details see Sudarikov [17] and references therein). Szidat [47] reared adult trematodes from metacercariae of *T. typica* from the gonads of two species of leech (*Erpobdella octoculata* and *Heamopis sanguisuga*) and also identified them as *Cotylurus cornutus*. These results prove the possibility of the occurrence of tetracotyles of *C. cornutus* in two, clearly distinct groups of intermediate host, i.e. snails and leeches. The identification of *T. typica* from leeches as *C. cornutus* confirmed the results of Timon-David [48] and Dobrowolski [3]. Using material collected from leeches, Szidat [47, 49] described for the first time another type of metacercariae, *Tetracotyle gracilis*, and identified the adult form as *Apatemon gracilis* (Rudolphi, 1819). During studies on the taxonomic composition of strigeid metacercariae from leeches of the Volga estuary, Sudarikov et al. [45] confirmed the validity of *T. gracilis* as the larval form of *A. gracilis* but questioned the status of *T. typica* from leeches as *C. cornutus*. In the opinion of these authors, *T. typica* from leeches represents another species that is closely related to *C. cornutus*. Sudarikov et al.’s conclusions [45] were further confirmed by Vojtek et al. [4], who also stated that tetracotyle larvae of *Cotylurus* from leeches probably belong to a closely related, but different species of *Cotylurus* than tetracotyle larvae from snails that are commonly accepted in the contemporary literature as *C. cornutus*. However, these hypotheses were never confirmed, and the issue of the identity and taxonomic position of *Cotylurus* metacercariae occurring in leeches and snails remained unresolved for years. According to the literature, the occurrence of tetracotyles of four species of *Cotylurus* has been detected in leeches: *C. cornutus*, *C. hebraicus* Dubois, 1934 *C. strigeoides* Dubois, 1958 and *C. szidati* Zazornova, 1991 [5, 50–53]. Among these, tetracotyles of two species (*C. cornutus*, *C. strigeoides*) were also recorded from snail intermediate hosts [5, 50]. However, their real taxonomic position remains doubtful because of the subtle morphological differences between the tetracotyle forms of *Cotylurus* [53]. Moreover, the taxonomic consequences of morphological variability observed among tetracotyles of *Cotylurus* have not been confirmed by molecular studies. For these reasons, recent knowledge concerning the morphology and taxonomic position of tetracotyles from snails and leeches should be considered to be highly insufficient and to require urgent verification using molecular studies based on well-determined reference materials from definitive hosts. Importantly, recent molecular studies have revealed unexpected high molecular diversity within *Cotylurus* [15, 32], including morphologically well-established species [14].

In the present study, metacercariae of *Cotylurus* detected in leeches were identified as two species, *C. strigeoides* and *C. syrius*, and this identification was confirmed by the parallel, comparative morphological and molecular analysis of adult specimens sampled from definitive avian hosts and GenBank sequences. Importantly, while leeches and snails are recognised as the second intermediate host for *C. strigeoides* [5, 53], the life cycle of *C. syrius* remains unknown. Our molecular identification of metacercariae detected in *Haemopis sanguisuga* from Gdańsk Pomerania as *C. syrius* for the first time clearly indicates the route of transmission of this strigeid to the avian definitive hosts by leeches and emphasises the importance of this invertebrate as a second intermediate host. Moreover, we consider the lack of tetracotyle forms of *Cotylurus cornutus* in leeches as truly surprising. In Poland, this strigeid species is one of the most important elements of the trematode fauna of anseriform birds [54]. The prevalence of *C. cornutus* in the population of mallard *Anas platyrhynchos* from Gdańsk Pomerania is about 20% (G. Kanarek, unpublished data), which could reflect the common occurrence of its invasive stages in the aquatic environment in the study area. On the other hand, according to results obtained by Schwelm et al. [55], the distribution of larval stages (cercariae) of *Cotylurus* sp. within snail intermediate hosts revealed site-dependent distribution, i.e. parasites can be present in some sampling sites, but absent in others. However, the study area in Gdańsk Pomerania (near the Gulf of Gdańsk and Vistula Lagoon) is recognised as a well-known refuge of water and wetland birds with its numerous large drainage canals with slow-flowing waters, constituting a strongly homogeneous habitat for local bird populations. Taking this information into account, the absence of metacercariae of *C. cornutus* in the helminth fauna of leeches can suggest that the invasive form of this trematode species does not use leeches as intermediate hosts. This observation, along with other recently obtained results (identification of *C. syrius* and *C. strigeoides* in the leech helminth fauna), supports the hypothesis previously formulated by Sudarikov et al. [45] and Vojtek et al. [4] that tetracotyles of *C. cornutus* occur only in snail intermediate hosts, while the tetracotyle form detected in leeches represents different species of *Cotylurus*. Moreover, the real species composition and molecular diversity within the genus *Cotylurus* that utilises various invertebrate hosts (snails and leeches) remains unknown. Additionally, the results presented here clearly indicate the separation of ecological niches and life cycles between some species of *Cotylurus*, with potential serious evolutionary consequences for a wide range of host–parasite relationships.

The results obtained in the present study clearly confirm the validity of the genus *Cotylurus*. All markers placed analysed members of this genus in one, well-supported clade, with high diversity within the clade. However, the observed phylogenetic relationships within *Cotylurus* varied in relation to the analysed loci, which, in our opinion, reflects the limited availability of fully reliable comparative sequences from related taxa in GenBank. Despite this, the sequenced specimens of *C. strigeoides* (both adults and tetracotyle) formed a well-supported separate clade that was particularly well defined in the phylogeny based on 28S rDNA loci (Fig. 2). In the analysis based on the ITS2 marker, isolates of *C. strigeoides* created a sister branch to a single isolate of *C. gallinulae* (JX978441) sampled from *Aythya affinis* in Mexico and provided by Hernández-Mena et al. [56] (Fig. 3). Heneberg et al. [14] suggested a high level of similarity between some lineages of *C. cornutus* (isolates from *Anas crecca* from the Czech Republic) and sequences *C. gallinulae* (JX978441), but our phylogeny, based on several new isolates and supplemented by GenBank data, did not confirm these assumptions. The current phylogeny based on ITS2 sequences (length 270 bp) revealed only 0.4% difference (1 nucleotide) between *C. gallinulae* (JX978441) from Mexico and recently obtained sequences of *C. strigeoides* (both adults and metacercariae), which strongly suggests that they are conspecific. Moreover, Locke et al. [32] suggested the identity of COI sequences of *Cotylurus strigeoides* sampled from *Aythya collaris* in Canada (MH581280-2) and *C. gallinulae* (JX977781) provided by Hernández-Mena et al. [56]. Importantly, both JX978441 and recently sequenced adult specimens of *C. strigeoides* were sampled from anatid birds, which are recognised as the typical final hosts for this trematode species, while *C. gallinulae* is recognised as a typical parasite of Rallidae. According to Locke et al. [32], this suggests the possibility of incorrect determination of the material from Mexico. Another interesting question related to the position of *C. gallinulae* is the validity of recently sequenced specimens of *Cotylurus hebraicus*. This species was first described on the basis of trematodes collected from the Eurasian coot *Fulica atra* sampled in Syria and further placed as a subspecies within *Cotylurus gallinulae* as *C. gallinulae hebraicus* together with *C. gallinulae gallinulae* (Lutz, 1928) Dubois, 1937, *C. gallinulae ban* Yamaguti, 1939 and *C. gallinulae vitellosus* Lumsden et Zischke, 1963, which suggests their close affinity [19]. All these taxa are recognised as typical parasites of coots, moorhens and rails, but with different geographical distributions. Some authors [57–59] have recognised *C. hebraicus* as a valid taxon with species rank, and this has been confirmed in a recently obtained phylogeny, but the real taxonomic position and validity of other subspecies remain to be established. In our opinion, given their morphological similarity and narrow range of hosts, subspecies within *C. gallinulae* should be treated as a synonym of *C. hebraicus*. Confirmation of this hypothesis requires a detailed analysis of strigeid trematodes collected from typical avian final hosts (Rallidae) from a wide geographic distribution and comparison with sequenced specimens of *C. hebraicus* and other *Cotylurus* taxa.

Our results reveal the unclear taxonomic position and composite structure of *C. syrius* and *C. cornutus*, as reported previously by Heneberg et al. [14]. According to these authors, trematode specimens morphologically identified as *C. syrius* and sampled from the typical host, the mute swan *Cygnus olor*, represent two distinct molecular lineages: one recognised as the typical *C. syrius *(*s.s.*), and the other, according to Heneberg et al. [14], as a *C. cornutus*-like isolate due to its similarity to sequences obtained from adult specimens of *C. cornutus* sampled from Eurasian teal *Anas crecca*. Recently obtained sequences of the COI and ITS2 loci obtained from metacercariae sampled from *Haemopis sanguisuga* from Gdańsk Pomerania and adult trematodes collected from a swan from Kraków are almost identical to sequences obtained by Heneberg et al. [14] from adult specimens of *C. syrius* (*s.s.*) (MF628093, MF628099 for ITS2; MF628057, MF628059 for COI) (Figs. 3, 4). The identity of the above-mentioned sequences was also confirmed using the 28S rDNA locus (Fig. 2). Additionally, sequences of the ITS2 fragment isolated from adult specimens of *C. syrius* sampled from *Cy. olor* from Lower Silesia were found to be closely related to the sequence reported by Heneberg et al. [14] (MF628091) and are described as *C. cornutus*-like isolates of *C. syrius* (Fig. 3). Phylogenetic reconstruction based on the COI locus and the GMYC analysis revealed a separate lineage clustering *C. cornutus*-like *C. syrius* with isolates of *C. cornutus* obtained from the typical definitive host, the mallard *Anas platyrhynchos* (Fig. 5). Sequences of the COI locus of *C. cornutus* obtained in the current study differed from sequences reported by Heneberg et al. [14] under the name *C. cornutus* (MF628064) and were almost identical to recently sequenced adult specimens and the tetracotyle of *C. strigeoides* (Fig. 4, 5). The obtained sequences supplemented with GenBank data clearly confirmed the existence of three distinct lineages within species morphologically identified as *C. syrius*: one lineage identified by Heneberg et al. [14] as *C. syrius* (*s.s.*), recorded from the definitive host *Cy. olor* from the Czech Republic and Poland (Kraków) and in the tetracotyle from the leech *H. sanguisuga* from Gdańsk Pomerania, and two lineages identified by Heneberg et al. [14] as *C. cornutus*-like *C. syrius*. One of these grouped some specimens of *C. syrius* sampled from a typical definitive host, the swan *Cy. olor*, from the Czech Republic and Poland (Lower Silesia), and the second, collected from *Cy. olor* from the Czech Republic. The COI locus of the latter is almost identical to that of *C. cornutus* collected from the typical definitive host, the mallard *A. platyrhynchos* from Poland (Vistula Lagoon). In this context, swans in central Europe can be parasitised by three (not 2, as suggested previously by Heneberg et al. [14]), morphologically indistinguishable but molecularly different species of *Cotylurus*, classified as *C. cornutus* or *C. syrius*, which strongly suggests that these lineages represent a complex of cryptic species. In this regard, our results clearly reveal the urgent need to verify the validity and range of morphological criteria enabling the identification of adult specimens of some *Cotylurus* species. Moreover, based on the obtained results, we cannot exclude that the observed situation is a consequence of separation of ecological niches and hosts of tetracotyle between *C. syrius* (*s.s.*) lineages (tetracotyle in leeches) and a *Cotylurus-*like *C. syrius* lineage (tetracotyle in snails).

Recently obtained data have revealed the separate position of *Cotylurus raabei* within *Cotylurus*. For many years, this species was placed in the genus *Cotylurus* [19], *Cotylurostrigea* Sudarikov, 1961 [27, 57] or *Strigea* [20, 26]. Based on the results of morphological and cladistics analyses, Zazornova and Sysoev [28] synonymised *Cotylurostrigea* with *Cotylurus* and placed *C. raabei* within the latter. In the phylogeny based on 28S rDNA and COI markers, the position of this species is separate, forming a sister branch to the other *Cotylurus* species (Figs. 2, 4), and *C. raabei* is clearly distinct, while in the phylogeny based on the ITS region this species is placed in a lineage between the recently sequenced *C. hebraicus* and isolates of cercarial *Cotylurus* sp. infecting *Biomphalaria straminea* in Brazil (MN179272 and MN179271) [16]. The ITS2 sequences (270 bp) from *C. raabei* differed by 4.8% from those of *C. hebraicus* and by 5.2% from those of *Cotylurus* sp. from Brazil (MN179272 and MN179271). Given the inconsistent results obtained from the analysis of different loci, the taxonomic status of *C. raabei* remains unexplained and awaits further studies.

Another problem that is rarely mentioned in the contemporary literature is the identification, genetic variability and taxonomic position of metacercariae of *Australapatemon* from leeches. Due to their morphological similarity, the metacercariae and adults of *Australapatemon* have for many years been erroneously confused with *Apatemon* (e.g. [6]). According to the literature, one of the features enabling fully reliable differentiation between the genera *Australapatemon* and *Apatemon* (other than the structure of excretory systems in cercariae) is the host of the invasive stages (fish in *Apatemon*, leeches in *Australapatemon*). However, this is not true in all cases. Negm-Eldin and Davies [60] revealed that metacercariae of *Apatemon hypseleotris* from Australia can develop in both leeches and fish. Considering that the structure of cercariae of *A. hypseleotris* is typical for genus *Australapatemon* (14 flame cells), the taxonomic position of this species should be elucidated in further analyses [6]. Another species of *Apatemon* with a doubtful taxonomic position and metacercariae in leeches is *Apatemon jamesi* [61]. On the other hand, all species of *Australapatemon* with described life cycles utilise leeches as the hosts of invasive stages [6, 10, 20, 62–65]. Unfortunately, studies on the molecular identification, taxonomic position and diversity of *Australapatemon* metacercariae occurring in leeches from various ecosystems are scarce [6, 12].

Similar to the phylogenetic relationships within the genus *Cotylurus* discussed above, the phylogenetic relationships among *Australapatemon* varied with the loci analysed, which again, in our opinion, reflects the limited availability of fully reliable comparative sequences of related taxa in GenBank. Our data illustrate the inconsistent and confusing taxonomic status of sequenced tetracotyles of *Australapatemon*. Regarding the 28S rDNA fragment, the results revealed the greatest similarity to the sequences of *Au. burti* from Mexico (MF398342—99.9%, 1-nucleotide difference) and *Au. niewiadomski* from New Zealand (KT334164, KT334165—99.4%, 6-nucleotide difference) (Fig. 2). These results are in partial disagreement with sequences of the ITS2 fragment recently sampled from metacercariae from leeches that showed 100% similarity with sequences described as *Au. minor* from *Anas platyrhynchos* from the Czech Republic (MF628095) and *Au. burti* from cercariae from Slovakia (KU950451) and the USA (KY570947). Moreover, to increase confusion, the sequences of ITS2 were also similar to those from a trematode identified as *Au. mclaughlini* (1-nucleotide difference compared to recently obtained isolates) and *Au. burti* from Mexico (2-nucleotide difference). The COI sequence analysis also gave inconclusive results: all sequences were placed in a separate clade, sister to *Au. minor* (MF628066) from *Anas platyrhynchos* from the Czech Republic (Fig. 4), and revealed high intraspecific diversity. Therefore, in the light of the most recent available molecular data, tetracotyle of *Australapatemon* represents, most probably, *Au. minor*. However, due to the lack of comparable molecular data (preferably multi-locus) derived from type localities of *Au minor* and *Au. burti*, the phylogenetic relationship between both species is still unclear. Therefore, we assume that the second scenario, where *Au. minor* is a junior synonym of *Au. burti*, is also possible. The taxonomic position of the trematode identified as *Australapatemon* sp. collected from *Anas strepera* from Vistula Lagoon is also ambiguous: recently obtained sequences of 28S rDNA showed 100% homology with previously published sequences of *Au. burti* from Canada (KY207625) [13] and Mexico (MF398342) [66]. Regarding the ITS2 fragment, our isolates presented the highest similarity with sequences of *Australapatemon* sp. (MK168687 and MK168688) from France [25]. Based on the COI locus, an isolate of *Australapatemon* sp. from *Anas strepera* creates a separate branch to all other *Australapatemon* sequences available in GenBank, indicating its separate position (Fig. 4).

The phylogeny based on 28 rDNA and ITS2 also revealed unclear relationships between *Au. burti* and *Au. minor*. *Australapatemon burti* (Miller, 1923) was originally described from North America and often misreported as *Apatemon gracilis* [17, 63, 67–69], and has recently been widely recorded from the Holarctic (central Europe) [23, 70, 71] and neotropical regions [6, 56, 72, 73], and references therein], but only few of these records outside North America have been confirmed by molecular analysis [23, 56]. However, these data strongly suggest the cosmopolitan distribution of this species. *Australapatemon minor* Yamaguti, 1933 is recognised as a typical parasite of Anseriformes in the Palearctic region [19, 74]. Therefore, the sympatric occurrence of these two species (*Au. burti* and *Au. minor*) in the territory of central Europe would not be surprising. Moreover, as mentioned above, the study area in Gdańsk Pomerania (near the Gulf of Gdańsk and Vistula Lagoon) is recognised as a well-known refuge of water and wetland birds. In addition to the rich and diversified fauna of breeding birds, these places are an important resting place for birds migrating mainly from the north and north-east [75], which creates the possibility of some species of helminths being brought into the area by migrating birds from nesting grounds located, for example, on the Scandinavian Peninsula or Siberia. As such, helminth taxa unusual for this geographical region could appear, especially in avian definitive hosts during migration. Such conditions mean that extreme caution is required in the interpretation of molecular data. Unfortunately, while fragments of 28S rDNA clearly revealed the similarity of recently obtained isolates from tetracotyle and adult *Austalapatemon* sp. with sequences of *A. burti*, sequences of ITS2 isolates from tetracotyle showed 100% homology with sequences described as *Au. minor* and *Au. burti*. The main underlying problem is that the only sequences of ITS2 and COI fragments of *Au. minor* available in GenBank and provided by Heneberg et al. [14] are rather short (270 bp for ITS2 and 295 bp for COI), which forced the shortening of our sequences and precluded the full comparison of these sequences with other data (based on full-length fragments). In phylogenetic analyses, fragments of this limited length have limited significance and reduce the reliability of any conclusions.

Our results reveal the discursive and inconsistent status of the genera *Apatemon* and *Australapatemon*. In the phylogenies based on 28S rDNA and ITS2, all *Australapatemon* sequences analysed were placed in one, well-supported clade, separate from other strigeid genera (*Cotylurus* and *Apatemon*), with the exception of sequences of *Apatemon fuligulae,* which were located between *Australapatemon* (Figs. 2, 3). In the phylogeny based on COI fragments, our sequences of *Ap. fuligulae* were almost identical to sequences of *Ap. fuligulae* submitted by Heneberg et al. [14] (MF628055), which confirmed the valid determination of this species. Importantly, both isolates were placed in a branch with *Australapatemon* species. The different generic position of *Ap. fuligulae* obtained in phylogenies based on various other loci may indicate the inappropriate generic status of this species. These suspicions were confirmed by the phylogeny based on 28S rDNA fragments: sequences of *Ap. fuligulae* were similar to Canadian sequences of *Australapatemon* sp. (MF124270, 3-nucleotide difference) provided by Gordy et al. [13] and isolated from an adult trematode specimen collected from the northern pintail *Anas acuta* and cercariae emerging from *Stagnicola elodes*. On the other hand, Yamaguti [76] detected the metacercariae of *Ap. fuligulae* (*Tetracotyle fuligulae*) in the skin and musculature of fish (*Parasilurus asotus*, *Pseudobagrus aurantiacus*) from Lake Biwa. Therefore, the taxonomic position of *Ap. fuligulae* should be considered as doubtful. Establishing its position will require further studies on both the genetic variability within this species and a detailed analysis of the morphology of cercariae and adults, as well as determination of the second intermediate host (fish or leeches) in the life cycle of *Ap. fuligulae*.

To summarise, in our opinion, the unclear and inconsistent status of *Apatemon* and *Australapatemon*, which became especially evident using the COI and ITS markers, results mainly from the invalid determination of sequenced adult and larval trematodes: from a morphological point of view, as mentioned in the "Introduction", the two genera are quite similar and often misidentified in the literature. Erroneous determination of sequenced adult and larval stages of both *Apatemon* and *Australapatemon* result in the erroneous description of the sequences deposited in GenBank, which increases the chaos in the phylogenies based on them. Based on our own experience concerning the morphology of these genera and their ecology, as well as the literature, both of which show key differences between the life cycles of *Apatemon* and *Austalapatemon*, we believe that these genera are valid and distinct, as confirmed by the 28S rDNA sequences. However, full confirmation of this assertion requires a meticulous review of all sequences of *Apatemon* and *Austalapatemon* deposited in GenBank, which is not possible without detailed resolution of the life cycles of particular species within these genera.

## Conclusions

Our study suggests that encysted and non-encysted tetracotyle metacercariae derived from leeches from Poland represent two separate strigeid genera: *Australapatemon* and *Cotylurus*. In light of currently available molecular data and the results from the present study, tetracotyle of *Australapatemon* most probaby represents *Au. minor*; however, unclear phylogenetic relationships between *Au. burti* and *Au. minor* reduce the reliability of this conclusion. Detected metacercariae of *Cotylurus* were identified as two species, namely *C. strigeoides* and *C. syrius*. Our ecological and molecular data suggest that tetracotyle of *C. cornutus* may occur mainly in snail intermediate hosts, while the tetracotyle form detected in leeches represents non-*C. cornutus* species. On this basis, we suggest the separation of ecological niches and life cycles between *C. cornutus* and *C. strigeoides*/*C. syrius* with potential evolutionary consequences for a wide range of host–parasite relationships.

## Supplementary Information

**Additional file 1: Table S1.** Primers used in the present study. **Table S2.** The list of sequences of digenean representatives available in GenBank used in the molecular analyses. *A* Adult, *C* cercaria, *M* metacercaria

## Data Availability

All data generated or analysed during this study are included in present article and additional files, all necessary sequences were deposited in GenBank database.

## Abbreviations

COI: Cytochrome* c* oxidase subunit 1
GMYC: Generalised Mixed Yule Coalescent model
GTR + I + G: General time reversible model with estimates of invariant sites and gamma distributed among-site variation
HKY + G: Hasegawa–Kishino–Yano substitution model with gamma distributed among-site variation
ITS: Internal transcribed spacer region
ITS2: Second internal transcribed spacer region
mtDNA: Mitochondrial DNA
rDNA: Ribosomal DNA
28S: Nuclear large ribosomal subunit gene
