# Supplementary material for: Leeches as the intermediate host for strigeid trematodes: genetic diversity and taxonomy of the genera Australapatemon Sudarikov, 1959 and Cotylurus Szidat, 1928
Source: Parasit Vectors. 2021 Jan 12;14:44. doi: 10.1186/s13071-020-04538-9 (PMC7805170; doi:10.1186/s13071-020-04538-9)
Supplement: Supplementary file 1 — Additional file 1: Table S1. Primers used in the present study. Table S2. The list of sequences of digenean representatives available in GenBank used in the molecular analyses. A Adult, C cercaria, M metacercaria [file 13071_2020_4538_MOESM1_ESM.docx]

**Table S1.** Primers used in the present study

| **Gene** | **Primer** | **Sequence** | **Thermocycling profile** | **Reference** |
| --- | --- | --- | --- | --- |
| 28S | digl2 | 5'-AAGCATATCACTAAGCGG-3' | 95°C/3 min — initial denaturation; 95°C/30 s, 50°C/30 s, 72°C/1 min — 35 cycles; 72°C/5 min— ﬁnal extension | [1] |
|  | 1500R | 5’-GCTATCCTGAGGGAAACTTCG-3’ |  |  |
| ITS1-5.8SrDNA-ITS2 | BD1 | 5'-GTCGTAACAAGGTTTCCGTA-3' | 94°C/5 min— initial denaturation; 94°C/30 s, 55°C/30 s, 72°C/1 min — 35 cycles; 72°C/10 min — ﬁnal extension | [2] |
|  | BD2 | 5'-TATGCTTAAATTCAGCGGGT-3' |  |  |
|  | NLF | 5′-TTTGyACACACCGCCCGTCG-3′ | 95°C/3 min — initial denaturation;  95°C/30 s, 48°C/30 s, 72°C/45 s —  40 cycles; 72°C/7min — ﬁnal extension | [3] |
|  | NLR | 5′-ATATGCTTAArTTCAGCGGGT-3′ |  |  |
|  | BD3 | 5′-GAACATCGACATCTTGAACG-3′ | 94°C/1 min— initial denaturation; 94°C/1 min,  50°C/1 min, 72°C /1 min — 35 cycles;  72°C/10 min — ﬁnal extension | [4] |
|  | BD4 | 5′-ATAAGCCGACCCTCGGC-3′ |  |  |
| COI | JB3 | 5′-TTTTTTGGGCATCCTGAGGTTTAT-3′ | 95°C/ 3 min — initial denaturation;  95°C/30 s,  48°C/30 s  72°C/45 s —  35 cycles;  72°C/7 min — ﬁnal extension | [5] |
|  | JB13 | 5′-TCATGAAAACACCTTAATACC-3′ |  |  |
|  |  |  |  |  |
|  | JB4.5 = 240 | 5′-TAAAGAAAGAACATAATGAAAATG-3′ | 95°C/5 min— initial denaturation; 94°C/40s,  55°C/50s,  72°C /2 min — 35 cycles;  72°C/10 min — ﬁnal extension | [6]  [7, 8] |
|  | CO1_Rtrema | 5′-CAACAAATCATGATGCAAAAGG-3′ |  |  |

**Table S2.** The list of sequences of digenean representatives available in GenBank used in the molecular analyses. Abbreviations: A - adult, C - cercaria, M - metacercaria

| **Digenean taxa** | **Host species and geographic origin** | **Life cycle stage** | **GenBank No** | | |
| --- | --- | --- | --- | --- | --- |
|  |  |  | **COI** | **28S** | **ITS** |
| *Alaria alata*  Schrank, 1788 | *Nyctereutes procyonoides*  Ukraine | A | - | AF184263 | - |
| *Apatemon annuligerum* (Nordmann, 1832) | *Perca fluviatilis*  United Kingdom | M | AJ314763 | - | - |
| *Apatemon annuligerum* (Nordmann, 1832) | *Perca fluviatile*  United Kingdom | M | - | - | AJ314762 |
| *Apatemon fuhrmanni*  Dubois, 1937 | *Cygnus olor*  Czech Republic | A | MF628060 | - | MF628092 |
| *Apatemon fuhrmanni*  (Dubois, 1937) | *Cygnus olor*  Czech Republic | A | MF628061 | - | - |
| *Apatemon fuhrmanni*  Dubois, 1937 | *Cygnus olor*  Czech Republic | A | - | - | MF628098 |
| *Apatemon fuhrmanni*  Dubois, 1937 | *Cygnus olor*  Czech Republic | A | - | - | MF628096 |
| *Apatemon fuligulae*  Yamaguti, 1933 | *Aythya ferina*  Czech Republic | A | MF628055 | - | - |
| *Apatemon gracilis* (Rudolphi, 1819) | *Oncorhynchus mykiss*  United Kingdom | M | AJ301894 | - | - |
| *Apatemon gracilis* (Rudolphi, 1819) | *Gasterosteus aculeatus*  Norway | M | - | KY513177 | - |
| *Apatemon gracilis* (Rudolphi, 1819) | *Oncorhynchus mykiss*  United Kingdom | M | - | - | AJ301893 |
| *Apatemon* sp. 'jamiesoni' | *Gobiomorphus cotidianus*  New Zealand | M | KT334182 | - | - |
| *Apatemon* sp. 'jamiesoni' | *Phalacrocorax punctatus*  New Zealand | A | - | KT334169 | - |
| *Apatemon* sp.  Szidat, 1928 | *Gasterosteus aculeatus*  Norway | M | - | KY513179 | - |
| *Australapatemon burti* (Miller, 1923) | *Anas diazi*  Mexico | A | - | MF398342 | - |
| *Australapatemon burti* (Miller, 1923) | *Anas americana*  Mexico | A | - | - | JX977785 |
| *Australapatemon burti* (Miller, 1923) | *Anas cyanoptera*  Mexico | A | - | - | JX977786 |
| *Australapatemon burti* (Miller, 1923) | *Oxyura jamaicensis*  Mexico | A | - | - | JX977788 |
| *Australapatemon burti* (Miller, 1923) | *Radix labiate*  Slovakia | C | - | - | KU950451 |
| *Australapatemon burti* (Miller, 1923) | *Helisoma trivolvis*  Canada | C | - | KY207625 | KY207626 |
| *Australapatemon mclaughlini*  Gordy et al., 2017 | *Physella gyrina*  Canada | C | - | - | KY207628 |
| *Australapatemon minor*  (Yamaguti, 1933) | *Anas platyrhynchos*  Czech Republic | A | MF628066 | - | MF628095 |
| *Australapatemon niewiadomski*  Blasco-Costa et al., 2015 | *Barbronia weberi*  New Zealand | M | KT334176 | KT334164 | - |
| *Australapatemon niewiadomski*  Blasco-Costa et al., 2015 | *Anas platyrhynchos*  New Zealand | A | - | KT334165 | - |
| *Australapatemon* sp. Sudarikov, 1959 | *Anas acuta*  Canada | A | - | MF124270 | - |
| *Australapatemon* sp. Sudarikov, 1959 | *Oxyura jamaicensis*  Canada | A | - | MF124269 | - |
| *Australapatemon* sp. Sudarikov, 1959 | *Oxyura jamaicensis*  Canada | A | - | - | KY570946 |
| *Australapatemon* sp. Sudarikov, 1959 | *Stagnicola palustris*  France | C | - | - | MK168688 |
| *Australapatemon* sp. Sudarikov, 1959 | *Stagnicola palustris*  France | C | - | - | MK168687 |
| *Australapatemon* sp. Sudarikov, 1959 | *Galba pervia*  China | M | - | - | KX781394 |
| *Australapatemon* sp. Sudarikov, 1959 | *Galba pervia*  China | C | - | - | KX781395 |
| *Bolbophorus damnificus*  Overstreet & Curran, 2002 | Ictalurus punctatus  USA | M | AF470602 | - | - |
| *Cotylurus cornutus* (Rudolphi, 1808) | *Anas crecca*  Czech Republic | A | MF628064 | - | - |
| *Cotylurus cornutus* (Rudolphi, 1808) | *Gyraulus acronicus*  Norway | M | - | KY513182 | - |
| *Cotylurus cornutus* (Rudolphi, 1808) | *Gyraulus acronicus*  Norway | M | - | KY513181 | - |
| *Cotylurus cornutus* (Rudolphi, 1808) | *Radix balthica*  Norway | M | - | KY513180 | - |
| *Cotylurus gallinulae*  (Lutz, 1928) | *Aythya affinis*  Mexico | A | - | - | JX977841 |
| *Cotylurus marcogliesei*  Locke et al. 2018 | *Lophodytes cucullatus*  Canada | A | - | MH521248 | MH521248 |
| *Cotylurus syrius*  Dubois, 1934 | *Cygnus olor*  Czech Republic | A | MF628056 | - | MF628091 |
| *Cotylurus syrius*  Dubois, 1934 | *Cygnus olor*  Czech Republic | A | MF628057 | - | MF628093 |
| *Cotylurus syrius*  Dubois, 1934 | *Cygnus olor*  Czech Republic | A | MF628059 | - | MF628099 |
| *Cotylurus syrius*  Dubois, 1934 | *Cygnus olor*  Czech Republic | A | MF628058 | - | - |
| *Cotylurus* sp.  Szidat, 1928 | *Biomphalaria straminea*  Brazil | M | - | - | MN179272 |
| *Cotylurus* sp.  Szidat, 1928 | *Biomphalaria straminea*  Brazil | C | - | - | MN179271 |
| *Tylodelphys excavata*  (Rudolphi, 1803) | *Planorbarius corneus*  Czech Republic | C | - | - | KC685364 |

**References**

1. Tkach VV, Littlewood DT, Olson PD, Kinsella JM, Świderski Z. Molecular phylogenetic analysis of the Microphalloidea Ward, 1901 (Trematoda: Digenea). Syst Parasitol. 2003;56:1-15.

2. Morgan JAT, Blair D. Nuclear rDNA ITS sequence variation in the trematode genus *Echinostoma*: an aid to establishing relationships within the 37-collar spine group. Parasitology 1995;111:609-615.

3. Van der Auwera G, Chapelle S, de Wachter R. Structure of the large ribosomal subunit RNA of *Phytophtora megasperma*, and phylogeny of the oomycetes. FEBS Lett. 1994;338:133-136.

4. Hernández-Mena DI, García-Prieto L, García-Varela M. Morphological and molecular differentiation of *Parastrigea* (Trematoda: Strigeidae) from Mexico, with the description of a new species. Parasitol Int. 2014;63:315-323.

5. Morgan JA, Blair D. Relative merits of nuclear ribosomal internal transcribed spacers and mitochondrial CO1 and ND1 genes for distinguishing among *Echinostoma* species (Trematoda). Parasitology 1998;116:289-297.

6. Heneberg P, Sitko J, Těšínský M, Rząd I, Bizos J. Central European Strigeidae Railliet, 1919 (Trematoda: Strigeidida): Molecular and comparative morphological analysis suggests the reclassification of *Parastrigea robusta* Szidat, 1928 into *Strigea* Abildgaard, 1790. Parasitol Int. 2018;67:688-701.

7. Bowles J, Blair D, McManus DP. Genetic variants within the genus *Echinococcus* identified by mitochondrial DNA sequencing. Mol Biochem Parasitol. 1992;54:165-73.

8. Mira O, Kuris AM, Torchin ME, Hechinger RF, Dunham EJ, Chiba S. Molecular genetic analyses reveal cryptic species of trematodes in the intertidal gastropod, *Batillaria* *cumingi* (Crosse). Int J Parasitol. 2005;35:793-801.
